# Multivalent nanoparticle-based vaccines protect hamsters against SARS-CoV-2 after a single immunization

**DOI:** 10.1038/s42003-021-02128-8

**Published:** 2021-05-19

**Authors:** Shiho Chiba, Steven J. Frey, Peter J. Halfmann, Makoto Kuroda, Tadashi Maemura, Jie E. Yang, Elizabeth R. Wright, Yoshihiro Kawaoka, Ravi S. Kane

**Affiliations:** 1 Influenza Research Institute, Department of Pathobiological Sciences, School of Veterinary Medicine, University of Wisconsin, Madison, WI USA; 2 School of Chemical & Biomolecular Engineering, Georgia Institute of Technology, Atlanta, GA USA; 3 Department of Biochemistry, University of Wisconsin, Madison, WI USA; 4 Cryo-EM Research Center, Department of Biochemistry, University of Wisconsin, Madison, WI USA; 5 Division of Virology, Department of Microbiology and Immunology, Institute of Medical Science, University of Tokyo, Tokyo, Japan

**Keywords:** Vaccines, Infectious diseases

## Abstract

The COVID-19 pandemic continues to wreak havoc as worldwide SARS-CoV-2 infection, hospitalization, and death rates climb unabated. Effective vaccines remain the most promising approach to counter SARS-CoV-2. Yet, while promising results are emerging from COVID-19 vaccine trials, the need for multiple doses and the challenges associated with the widespread distribution and administration of vaccines remain concerns. Here, we engineered the coat protein of the MS2 bacteriophage and generated nanoparticles displaying multiple copies of the SARS-CoV-2 spike (S) protein. The use of these nanoparticles as vaccines generated high neutralizing antibody titers and protected Syrian hamsters from a challenge with SARS-CoV-2 after a single immunization with no infectious virus detected in the lungs. This nanoparticle-based vaccine platform thus provides protection after a single immunization and may be broadly applicable for protecting against SARS-CoV-2 and future pathogens with pandemic potential.

## Abstract (web summary)

Chiba et al. describe the use of MS2 bacteriophage coat proteins to develop nanocarriers that display the SARS-CoV-2 spike proteins multivalently for vaccine applications. The vaccine elicited high neutralizing antibody titers and protected Syrian hamsters from virus infection after a single immunization.

## Introduction

The COVID-19 pandemic continues to rage worldwide with more than a million estimated fatalities already and global economic costs in the hundreds of billions of dollars. Without an effective vaccine, SARS-CoV-2 will continue to strain the world’s economies and devastate many facets of our society. Various vaccines such as nucleic acid-based vaccines, viral vector-based vaccines, subunit vaccines, and inactivated vaccines are in different stages of clinical trials^1^. The contemporary vaccine candidates focus on stimulating protective immune responses to the spike (S) protein of SARS-CoV-2, the protein that facilitates viral entry by binding to the angiotensin-converting enzyme 2 (ACE2) receptor on the surface of host cells^2^. Neutralizing antibodies that target the spike protein could, therefore, play a role in protecting the host from this viral infection^3,4^.

Although these standard vaccine platforms may provide the first generation of vaccines against SARS-CoV-2, nanotechnology^5^ has the potential to offer new and improved vaccine platforms against diseases caused by emerging viruses including SARS-CoV-2. Nanoparticles such as virus-like particles (VLPs) are ideal scaffolds for antigen display, because they emulate many of the properties of natural viruses including their size and geometry^5–8^. Moreover, the multivalent display of antigens from nanoscale scaffolds can result in the effective clustering of B cell receptors and greatly enhance their immunogenicity^9^. Indeed, a recent report confirmed that the S protein displayed on a nanoscale scaffold was more immunogenic in mice than the S protein administered alone, but the study used two sequential immunizations (prime + boost) and did not test protective efficacy in mice challenged with SARS-CoV-2^10^. In the present study, we created a general platform for nanoparticle-based antigen display that could provide protection against SARS-CoV-2 after a single immunization.

## Results and discussion

### Generation and in vitro characterization of nanoparticle-based vaccines

**Fig. 1:**
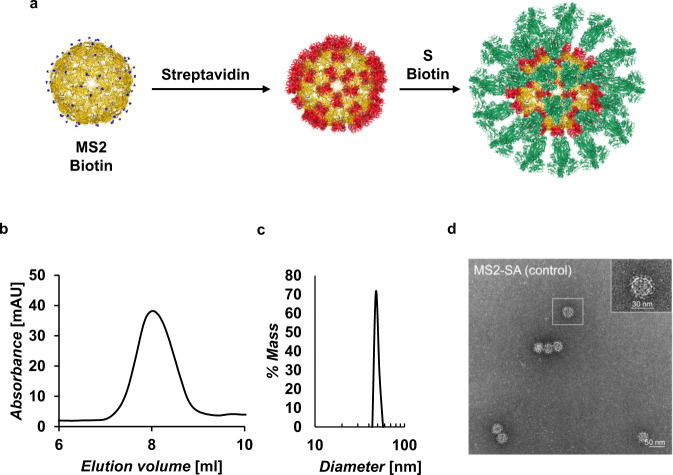
Assembly of VLP-S and characterization of MS2-SA VLP. **a** Scheme illustrating assembly of VLP-S, where biotinylated MS2 (yellow, PDB: 2MS2) is added to streptavidin (red, PDB:3RY2) to create the VLP. S (green, PDB: 6VSB) biotinylated at the C-terminus is mixed with the VLP to create VLP-S. Biotinylated residues are colored blue. **b** Size exclusion chromatography trace for MS2-SA VLP. The column void volume is 7.2 mL. **c** Characterization of the MS2-SA VLP by dynamic light scattering. **d** Negative-stain transmission electron micrograph of MS2-SA VLPs.

We first sought to develop a general platform for the VLP-based multivalent display of the S protein of SARS-CoV-2. VLPs comprise coat proteins that self-assemble to form repetitive, dense arrays of antigen that emulate the size and geometry of natural viruses^6^. We generated VLPs coated with streptavidin (SA) that display biotinylated antigens, such as biotinylated SARS-CoV-2 S protein (Fig. 1a), based on the very high-affinity biotin-streptavidin interaction.

Specifically, we generated VLPs based on the coat protein of the RNA bacteriophage MS2^7,11^. MS2 consists of 180 monomeric coat proteins that self-assemble to form an icosahedral structure consisting of 90 homodimers. Peabody et al. generated a variant of the MS2 coat protein in which the two subunits of the dimer were genetically fused and found that a surface loop on this single-chain dimer could tolerate the insertion of a peptide^12^. Accordingly, we generated a single-chain MS2 coat protein dimer wherein the second monomer had an AviTag inserted in this surface loop (Supplementary Note 1). The inserted AviTag allows for site-specific biotinylation by the enzyme BirA. DNA encoding this MS2-AviTag construct was co-expressed with BirA in BL21(DE3) competent Escherichia coli (E. coli) cells. Following expression, the cells were lysed and the MS2-AviTag was purified by using HiScreen Capto Core 700 columns and size exclusion chromatography (SEC). The purified MS2-AviTag was partially biotinylated due to its co-expression with BirA. A commercially available kit was then used to further biotinylate the MS2-AviTag in vitro, which resulted in near 100% biotinylation. The MS2-Biotin was then added dropwise to an excess of SA, which had been expressed as inclusion bodies, refolded, and purified using Iminobiotin Affinity Chromatography (IBAC). The resulting MS2-SA VLPs were separated from the excess SA through SEC. Sodium dodecyl sulfate-polyacrylamide gel electrophoresis (SDS-PAGE) was used to determine that each purified MS2-SA VLP contained ~72 streptavidin molecules (Supplementary Fig. 1). The purified MS2-SA VLPs were further characterized by analytical SEC (Fig. 1b), dynamic light scattering (DLS) (Fig. 1c), and negative-stain transmission electron microscopy (NS-TEM) (Fig. 1d). Characterization by DLS indicated that the purified MS2-SA VLPs were ~50 nm in diameter, whereas characterization by NS-TEM indicated uniform particles with a diameter of ~30 nm. The larger average size indicated by DLS may arise because the scattering intensity is proportional to the sixth power of the radius, resulting in a disproportionately higher weighting to larger particles.

**Fig. 2:**
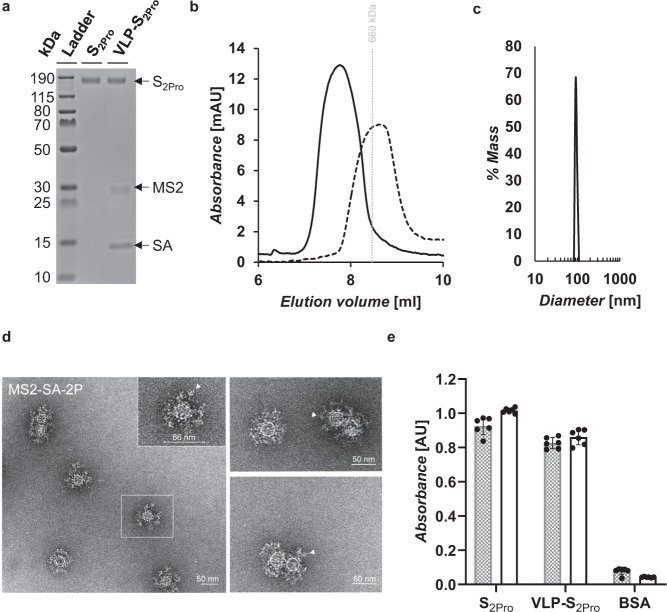
Characterization of S2Pro and VLP-S2Pro. **a** SDS-PAGE characterization of S_2Pro_ and VLP-S_2Pro_. The VLP-S_2Pro_ has been boiled to disrupt the streptavidin-biotin conjugation. The unprocessed gel is shown in Supplementary Fig. 3. **b** Size exclusion chromatography traces for S_2Pro_ (dashed line) and VLP-S_2Pro_ (solid line). The vertical gray line represents the peak elution volume of the molecular weight standard thyroglobulin (660 kDa). The column void volume is 7.2 mL. **c** Characterization of the VLP-S_2Pro_ (solid line) by dynamic light scattering. **d** Negative-stain transmission electron micrographs of S_2Pro_ incorporated on the surface of MS2-SA VLPs. Arrowheads (white) indicate the S_2Pro_ proteins on the VLP surface. **e** Characterization of the binding of Fc-ACE2 (gray) and CR3022 (white) to S_2Pro_ and VLP-S_2Pro_ by ELISA (mean ± SD, *n* = 6: two independent assays, each with three technical replicates). Source data are provided in Supplementary Data 1.

We next generated a biotinylated variant of the SARS-CoV-2 S protein that could be displayed on the MS2-SA VLPs. Wrapp et al. recently reported a prefusion-stabilized variant of the SARS-CoV-2 S protein, S-2P, which contains 2 proline substitutions^13^. To make a version of this variant that was compatible with display on the MS2-SA VLPs, we created plasmids encoding the stabilized prefusion S ectodomain with a C-terminal AviTag and a his-tag, which we termed S_2Pro_ (Supplementary Note 2). The AviTag allows biotinylation and subsequent conjugation to the VLPs, whereas the his-tag allows purification by use of immobilized metal affinity chromatography (IMAC). We expressed the S_2Pro_ protein in Expi293F cells and purified the secreted protein from the cell culture media by using IMAC. The protein was then biotinylated enzymatically in vitro by BirA. Finally, the protein was separated from BirA and other impurities by using SEC and the purity was characterized by use of SDS-PAGE (Fig. 2a and Supplementary Fig. 2).

The purified, biotinylated S_2Pro_ protein was then mixed with the MS2-SA VLPs to form VLP-S_2Pro_. SDS-PAGE was used to determine that each purified VLP-S_2Pro_ particle contained ~18 S_2Pro_ molecules (Supplementary Fig. 1). Further SDS-PAGE analysis of the VLP-S_2Pro_ revealed the expected three distinct bands (Fig. 2a): the upper band runs alongside S protein alone and appears at ~140 kDa, which corresponds to the approximate molecular weight of a single monomer of the S trimer; the middle band appears at the molecular weight of an MS2 coat protein dimer (~29 kDa); and the lower band corresponds to the molecular weight of a monomer of SA (~14 kDa). This characterization indicates that the VLP-S_2Pro_ is pure and consists of only S_2Pro_, SA, and MS2. The VLP-S_2Pro_ construct was also characterized by using analytical SEC (Fig. 2b). The UV trace of the VLP-S_2Pro_ is represented by a solid line, which appears as a single peak with no trailing shoulder. The lack of a trailing shoulder suggests that there is little to no unbound S_2Pro_ protein in the VLP-S_2Pro_ solution, as the UV trace of the S_2Pro_ protein alone results is a single peak that slightly trails the peak of the VLP-S and is represented by a dashed line. Furthermore, the locations of the peaks are consistent with the constructs’ size relative to the size of the molecular weight standard thyroglobulin (660 kDa). The location at which thyroglobulin elutes is represented by a vertical gray line.

We characterized the VLP-S_2Pro_ constructs by DLS (Fig. 2c) and then by NS-TEM (Fig. 2d) to confirm the presence and coating efficiency of biotinylated S_2Pro_ on the VLP. Consistent with biochemical characterization, VLP-S_2Pro_ displayed clear three-component layers, from outside to inside, prefusion-stabilized variants of S_2Pro_, SA, and MS2 (Fig. 2d). Compared to the naked MS2-SA, glycoprotein S_2Pro_ decorates the exterior of VLP- S_2Pro_ (Fig. 2d, white arrowheads), forming a ~20 nm layer of a spike-containing protein shell. This result is consistent with expectations, as the S protein (with the trimerization domain and C-terminal AviTag) would theoretically be ~20 nm in length. Finally, to ensure that the S proteins remained properly folded after conjugation to the VLPs, we assessed the binding of ACE2-Fc and the receptor-binding domain (RBD)–binding monoclonal antibody CR3022 to S_2Pro_ protein alone and to VLP-S_2Pro_ (Fig. 2e). ACE2 is the cellular receptor for SARS-CoV-2 and binds to the receptor-binding motif of the S protein^14^. A common mechanism of SARS-CoV-2 neutralization is the inhibition of S protein binding to ACE2, so it is important to demonstrate that the ACE2 binding site is properly folded^15,16^. CR3022 is an antibody that binds to the S protein RBD outside of the ACE2 binding site^15,17^. ELISA showed that both ACE2-Fc and CR3022 can bind to the S_2Pro_ protein alone and to VLP-S_2Pro_. This analysis demonstrates that the protein epitopes needed to elicit a neutralizing immune response to SARS-CoV-2 are correctly folded and accessible.

**Fig. 3:**
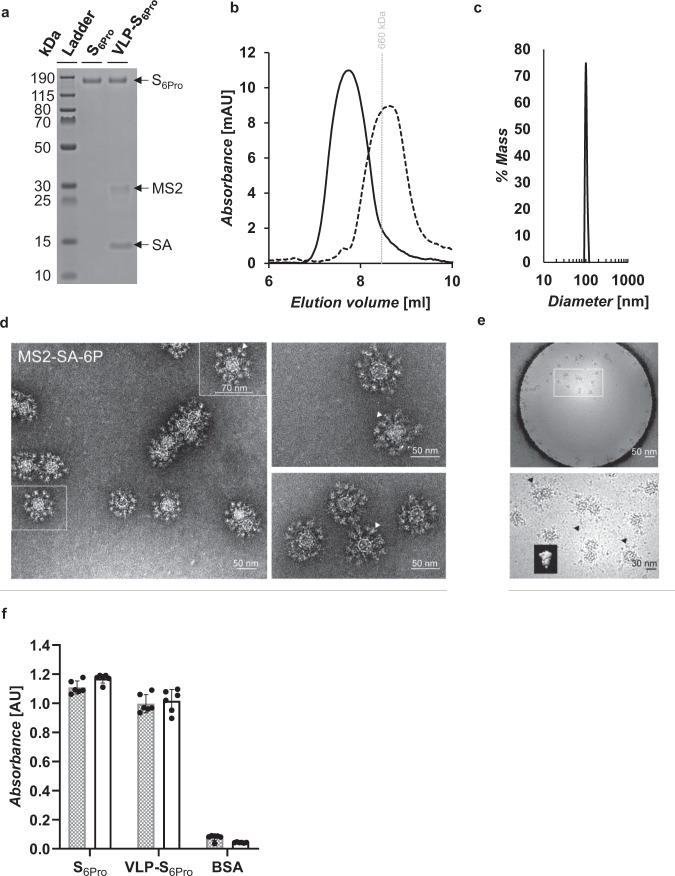
Characterization of S6Pro and VLP-S6Pro. **a** SDS-PAGE characterization of S_6Pro_ and VLP-S_6Pro_. The VLP-S_6Pro_ has been boiled to disrupt the streptavidin-biotin conjugation. The unprocessed gel is shown in Supplementary Fig. 3. **b** Size exclusion chromatography traces for S_6Pro_ (dashed line) and VLP-S_6Pro_ (solid line). The vertical gray line represents the peak elution volume of the molecular weight standard thyroglobulin (660 kDa). The column void volume is 7.2 mL. **c** Characterization of the VLP-S_6Pro_ (solid line) by dynamic light scattering. **d** Negative-stain transmission electron micrographs of S_6Pro_ incorporated on the surface of MS2-SA VLPs. Arrowheads (white) indicate the S_6Pro_ proteins on the VLP surface. **e** Cryo-EM of vitrified VLP-S_6Pro_. The inset shows a low-pass filtered to 10 Å volume of HexaPro structure (EMD: 22221, reported previously) for comparison. Arrowheads (black) indicate the representative S_6Pro_ proteins on the VLP surface. **f** Characterization of the binding of Fc-ACE2 (gray) and CR3022 (white) to S_6Pro_ and VLP-S_6Pro_ by ELISA (mean ± SD, *n* = 6: two independent assays, each with three technical replicates). Source data are provided in Supplementary Data 1.

We also generated VLPs displaying multiple copies of a second prefusion-stabilized variant of the S protein, called HexaPro, which was reported by Hsieh et al. to be more stable than S-2P and give a higher expression yield^18^. We expressed a variant of HexaPro containing a C-terminal AviTag and a his-tag, which we termed S_6Pro_ (Fig. 3a) (Supplementary Note 3). VLP-S_6Pro_ were generated and characterized (Fig. 3 and Supplementary Fig. 1) as described above for VLP-S_2Pro_. In addition, to preserve the sample’s native integrity, minimize conformational changes possibly introduced during the negative stain process, and further confirm the incorporation of spike proteins, we performed cryo-electron microscopy (cryo-EM) on the VLP-S_6Pro_ constructs. The MS-SA core was an approximately icosahedral sphere 30 nm in diameter and S_6Pro_ spikes were studded on the core and formed the outer shell (Fig. 3e), the morphology of which was comparable to the previous reported structure of S_6Pro_ (EMD: 22221^18^).

### Protective efficacy and antibody response to a single immunization in Syrian hamsters

**Fig. 4:**
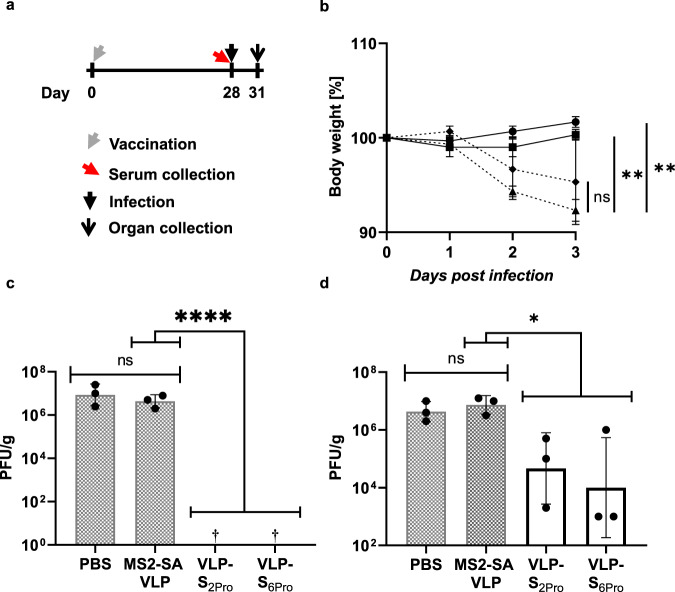
Protective efficacy of VLP-S. **a** Schedule for vaccination of hamsters, serum collection, infection, and organ collection. **b** Body weight of hamsters immunized with a single dose of either VLP-S_6Pro_ (solid line with circles), VLP-S_2Pro_ (solid line with squares), MS2-SA VLP (dashed line with triangles), or PBS (dashed line with diamonds) after SARS-CoV-2 infection (mean ± SD, *n* = 3 hamsters). ns: not statistically significant, **p < 0.01, determined by a one-way analysis of variance (ANOVA) and Dunnett post-hoc multiple comparison between groups (α = 0.1). Assumptions of the normality of residuals and homogeneity of variance were validated by the D’Agostino-Pearson test and the Brown–Forsythe test, respectively. **c** Viral titer in the lungs of hamsters immunized with either PBS, MS2-SA VLP, VLP-S_2Pro_ or VLP-S_6Pro_ three days after SARS-CoV-2 infection (geometric mean with geometric SD, *n* = 3 hamsters). †—No infectious virus was detected in the lungs of hamsters immunized with VLP-S_2Pro_ or VLP-S_6Pro_ (detection limit 10 PFU/g). ns: not statistically significant, ****p < 0.0001, determined by a one-way analysis of variance (ANOVA) and Dunnett post-hoc multiple comparison between groups (α = 0.1). Assumptions of the normality of residuals and homogeneity of variance were validated by the Shapiro-Wilk test and the Brown-Forsythe test, respectively. **d** Viral titer in the nasal turbinates of hamsters immunized with either PBS, MS2-SA VLP, VLP-S_2Pro_ or VLP-S_6Pro_ three days after SARS-CoV-2 infection (geometric mean with geometric SD, *n* = 3 hamsters). ns: not statistically significant, *p < 0.1, determined by a one-way analysis of variance (ANOVA) and Dunnett post-hoc multiple comparison between groups (α = 0.1). Assumptions of the normality of residuals and homogeneity of variance were validated by the Shapiro–Wilk test and the Brown–Forsythe test, respectively. Source data for Fig. 4b–d are provided in Supplementary Data 1.

We next evaluated the antibody responses elicited by these nanoparticle-based vaccine candidates in Syrian hamsters. We^19^ and others^20^ have demonstrated that Syrian hamsters are highly susceptible to SARS-CoV-2 infection and present with pathological phenotypes similar to those of infected humans, making hamsters an ideal animal model to evaluate vaccine candidates. Hamsters (four groups; three animals/group) were immunized with VLP-S_2Pro_, VLP-S_6Pro_, MS2-SA VLPs alone, or PBS along with Alhydrogel, an aluminum hydroxide base adjuvant. The hamsters were bled 28 days after immunization to characterize their antibody responses (Fig. 4a). Hamsters immunized once with the VLP-S conjugates had appreciable levels of IgG antibodies against the RBD of the S protein as determined by ELISA, with endpoint titers ranging from 2.6 × 10^4^ to 8.2 × 10^4^ and high neutralizing antibody titers (representing the reciprocal of the highest dilution that completely prevented cytopathic effects) ranging from 320 to 640 (Table 1). In contrast, as expected, negligible anti-S antibodies were detected in hamsters immunized with the controls (VLPs alone or PBS). We have compared these titers with some previously published reports. We stress, however, that the assays used in the literature are not standardized and some reports have even used different animal models, and the differences must therefore be interpreted with caution. Tostanoski et al.^21^ characterized the immunogenicity of adenovirus serotype 26 (Ad 26) vector-based vaccines expressing a stabilized SARS-CoV-2 S protein in hamsters. Ad26-S.PP, also termed Ad26.COV2.S, which has been evaluated in clinical trials, showed median endpoint antibody titers against the S RBD of up to 4757 four weeks after the first immunization. While neutralization assays used a pseudovirus, the median neutralizing antibody half-maximal inhibitory concentration (IC_50_) titers reported for the Ad26-S.PP were as high as 375. Corbett et al.^22^ conducted a study in mice to test the mRNA vaccine called mRNA-1273, for which they reported geometric mean endpoint titers of 4479 (against S) 4 weeks after a single dose. Neutralizing antibody reciprocal IC_50_ geometric mean titers against a pseudovirus 4 weeks after a single immunization with mRNA-1273 at the highest dose (10 μg) were 775. Thus, our endpoint and neutralizing antibody titers compare well with those obtained using these other modalities that have been through clinical trials.

**Table 1:** Antibody responses to single immunization in Syrian hamsters.

		RBD IgG endpoint titer^a^					Neutralizing antibody titer^b^				
Vaccine group	Animal #	Replicate	Replicate	Replicate	Geometric mean	Geometric SD factor	Replicate	Replicate	Replicate	Geometric mean	Geometric SD factor
		1	2	3			1	2	3		
PBS	1	<10	<10	<10			<10	<10	<10		
	2	<10	<10	<10	<10	–	<10	<10	<10	<10	–
	3	<10	<10	<10			<10	<10	<10		
MS2-SA VLP	1	<10	<10	<10			<10	<10	<10		
	2	<10	<10	<10	<10	–	<10	<10	<10	<10	–
	3	<10	<10	<10			<10	<10	<10		
VLP-S2_Pro_	1	20,480	40,960	20,480			320	640	320		
	2	81,920	40,960	81,920	35,113	1.78	640	320	320	373	1.36
	3	20,480	20,480	40,960			320	320	320		
VLP-S_6Pro_	1	81,920	81,920	81,920			640	640	640		
	2	81,920	81,920	81,920	70,225	1.36	640	640	640	549	1.36
	3	40,960	81,920	40,960			320	640	320		

^a^Viral antibody endpoint titers against the RBD (receptor-binding domain) from three independent assays (three animals in each group). Endpoint titers using 2-fold diluted sera were expressed as the reciprocal of the highest dilution with an optical density at 490 nm cutoff value >0.15; sera were collected on day 28 after immunization.

^b^Viral neutralization titers from three independent assays (three animals in each group). Endpoint titers using twofold diluted sera were expressed as the reciprocal of the highest dilution that completely prevented cytopathic effects; sera were collected on day 28 after immunization.

Four weeks after immunization, the animals were intranasally inoculated with 10^3^ plaque-forming units of SARS-CoV-2/UT-NCGM02/Human/2020/Tokyo^19^. While the animals in both control groups experienced significant weight loss, those immunized with VLP-S_2Pro_ had recovered their average initial weight by day 3, and those immunized with VLP-S_6Pro_ showed a slight increase in body weight over this 3-day period (Fig. 4b).

Three days after virus challenge, which is when virus levels in the lungs peak^19^, the animals were sacrificed and lung and nasal turbinate samples were collected. As expected, animals in both control groups (PBS and MS2-SA VLPs) had high viral loads in the lungs; however, in hamsters immunized with VLP-S_2Pro_ or VLP-S_6Pro_ no infectious virus was detected in the lungs (Fig. 4c). The lack of infectious virus in the lungs was consistent with the differences observed in body weight change between the vaccine and control groups. Moreover, despite the intranasal mode of challenge with SARS-CoV-2, the hamsters immunized with VLP-S_2Pro_ or VLP-S_6Pro_ had less virus in their nasal turbinates (Fig. 4d), with mean titers more than 150-fold lower (VLP-S_2Pro_) and more than 700-fold lower (VLP-S_6Pro_) relative to MS2-SA VLP controls.

In summation, we have developed a highly effective nanoparticle-based vaccine that provides protection in an animal model against SARS-CoV-2 after a single immunization. While these results are exciting, it is important to consider obstacles that might arise during clinical translation. One potential concern with subunit vaccines is the expression yield. Hsieh et al. designed the S_6Pro_ variant by introducing substitutions that improve both expression yield and stability and reported a yield of 10.5 mg/L in FreeStyle 293-F cells^18^. Expression in insect cells could also be evaluated for improving the expression yield. Further optimization would undoubtedly be needed to develop a cGMP (current Good Manufacturing Practices) compliant manufacturing process. Another potential concern is an immune response to the MS2-SA VLP scaffold. Antibody responses have been reported to other scaffolds, including viral vectors, and are certainly possible with our construct. The concerns are mitigated in part by the protective efficacy after a single dose. However, if necessary, the scaffold could be further shielded from the immune system by using techniques such as nanopatterning^23^. Another important issue is the emergence of “variants of concern”. In this context, vaccine platforms that generate a robust immune response would be advantageous as they might still be able to provide some protection against resistant strains despite a reduction in neutralizing antibody titers. Our vaccine platform could also be readily adapted for the display of S proteins from variant strains. In the future, it would be particularly important to use this platform to display engineered antigens that provide broader—pan-sarbecovirus or pan-coronavirus—immunity. Given the number of people that must be immunized, and societal habits, an ideal vaccine against SARS-CoV-2 would offer protection after only one immunization. The development of multiple effective vaccine platforms^21,24,25^ that can offer such protection is critical because vaccines remain the best approach for protection from current and future pandemics. The nanoparticle-based vaccine platform described here should be broadly applicable for protecting against important pathogens including, but not limited to, SARS-CoV-2 and influenza viruses.

## Methods

### Expression and purification of MS2

DNA encoding single-chain MS2 coat protein dimer with an AviTag inserted between the 14 and 15 residues of the second coat protein monomer was cloned into pET-28b between the NdeI and XhoI restrictions sites by GenScript Biotech Corporation (Piscataway, NJ). The MS2 dimer with the inserted AviTag was co-transformed with pAcm-BirA (Avidity LLC) into BL21(DE3) competent E. coli (New England Biolabs) according to the manufacturer’s instructions. The transformation was added to 5 mL of 2xYT media and grown overnight at 37 °C. The 5-mL starter culture was then added to 1 L of 2xYT media, which was incubated shaking at 37 °C until induction with IPTG (1 M; GoldBio) at an OD of 0.6. Immediately after induction, biotin (50 µM) was added to the culture and the incubator temperature was reduced to 30 °C. After overnight incubation, the culture was centrifuged for 7 min at 7000×*g* and the supernatant was decanted. The cell pellet was homogenized into 25 mL of 20 mM Tris Base (pH 8.0) supplemented with lysozyme (0.5 mg/mL; Alfa Aesar), a protease inhibitor tablet (Sigma-Aldrich), and benzonase (125 units; EMD Millipore). The resuspended cells were then kept on ice and stirred intermittently for 20min. Sodium deoxycholate (Alfa Aesar) was then added to a final concentration of 0.1% (w/v), and the mixture was sonicated for 3 min at 35% amplitude with a pulse of 3 s on and 3 s off (Sonifier S-450, Branson Ultrasonics). The sonication was repeated after allowing the lysate to cool on ice for 2 min. Next, the lysed cells were centrifuged for 30 min at 27,000 × *g*. The supernatant was collected, and centrifuged again for 15 min at 12,000 × *g*. The resulting supernatant was then diluted 3-fold in 20 mM Tris Base and filtered with a 0.45-µm bottle-top filter (VWR). Then, 25 mL of the diluted lysate was loaded onto four HiScreen Capto Core 700 columns (Cytiva) in series using an AKTA start system. The columns were washed with ~3 column volumes of 20 mM Tris Base while fractions were collected. Fractions were subsequently analyzed for purity and recovery of MS2 by using SDS-PAGE. Desirable fractions were pooled, concentrated by using a 10 kDa MWCO centrifugal filter (Millipore Sigma), and further purified by using a Superdex 200 Increase 10/300 column (Cytiva). MS2 was quantified by using a bicinchoninic acid assay (BCA) (Thermo Scientific).

### Expression, refolding, and purification of streptavidin (SA)

SA was expressed, refolded, and purified essentially as previously described^26,27^. Briefly, DNA encoding SA (Addgene plasmid #46367, a gift from Mark Howarth)^26^ was transformed into BL21(DE3) cells (New England Biolabs) according to the manufacturer’s protocol. The transformation was split among four culture tubes each containing 5 mL of 2xYT media, which were incubated overnight at 37 °C. Each 5 mL culture was added to one of four 1 L flasks of 2xYT and grown at 37 °C. Upon reaching an OD of 0.6, expression of inclusion bodies was induced using IPTG (1 M; GoldBio) and the temperature of the incubator was reduced to 30 °C. After incubation overnight, the culture was centrifuged for 7 min at 7000 × *g* such that 4 l of culture resulted in two cell pellets. Each pellet was resuspended in 50 mL of resuspension buffer (50 mM Tris, 100 mM NaCl, pH 8.0) supplemented with lysozyme (1 mg/mL; Alfa Aesar) and benzonase (500 units; EMD Millipore) and was allowed to incubate at 4 °C for 1 h with occasional mixing. These mixtures were then homogenized, brought to a concentration of 0.1% (w/v) sodium deoxycholate (Alfa Aesar), and sonicated (Sonifier S-450, Branson Ultrasonics) for 3 min at 35% amplitude with a pulse of 3 seconds on and 3 seconds off. The resulting lysate was then centrifuged for 15 min at 27,000 × *g*. The supernatant was discarded, and the two pellets were each again resuspended in 50 mL of resuspension buffer supplemented with lysozyme (1 mg/mL; Alfa Aesar) and the lysis procedure was repeated. This procedure resulted in two inclusion body pellets, which were then washed. Each inclusion body pellet was resuspended in 50 mL of wash buffer #1 (50 mM Tris, 100 mM NaCl, 100 mM EDTA, 0.5% (v/v) Triton X-100, pH 8.0), homogenized, and sonicated for 30 seconds at an amplitude of 35%. Each mixture was then centrifuged at 27,000 × *g* for 15 min and the supernatant was discarded. This wash was repeated twice. The two inclusion body pellets resulting from the third round of the initial wash were each resuspended in 50 mL of wash buffer #2 (50 mM Tris, 10 mM EDTA, pH 8.0), homogenized, and sonicated for 30 s at an amplitude of 35%. Each mixture was then centrifuged at 15,000 × *g* for 15 min. This wash was repeated once. The two resulting washed inclusion body pellets were then completely unfolded by resuspension in 10 mL of a 7.12 M guanidine hydrochloride solution. This mixture was stirred at room temperature for 1 h, and subsequently centrifuged at 12,000 × *g* for 10 min. The supernatant was drawn into a syringe, which was loaded onto a syringe pump, and added at a rate of 30 mL/h to 1 L of chilled PBS that was being stirred rapidly. This solution of refolded protein was stirred continuously overnight at 4 °C. Insoluble protein was then pelleted by centrifugation at 7000 × *g* for 15 min and discarded. The supernatant containing the folded SA was filtered by using a 0.45-µm bottle-top filter. The resulting filtrate was stirred vigorously, and ammonium sulfate was slowly added to a concentration of 1.9 M to precipitate out protein impurities. After being stirred for 3 h at 4 °C, the precipitate was removed by centrifugation for 10 min at 7000 × *g*. The supernatant was then filtered by using a 0.45-µm bottle-top filter. The ammonium sulfate concentration of the resulting filtrate was brought up to a total concentration of 3.68 M and stirred for 3 h at 4 °C to precipitate the SA. The SA precipitate was pelleted by centrifugation at 7000 × *g* for 20 min, and resuspended in 20 mL of Iminobiotin Affinity Chromatography (IBAC) binding buffer (50 mM Sodium Borate, 300 mM NaCl, pH 11.0). This SA solution was then passed through 5 mL of Pierce Iminobiotin Agarose (Thermo Scientific) in a gravity flow column (G-Biosciences) that had been pre-equilibrated with 5 column volumes of IBAC binding buffer. The IBAC column containing the bound SA was then washed with 20 column volumes of IBAC binding buffer. Then, 8 column volumes of elution buffer were passed through the column. The eluate was collected, dialyzed into PBS, and concentrated using a 10 kDa MWCO centrifugal filter (Millipore Sigma). SA was quantified by measuring the UV absorption at 280 nm.

### Assembly and purification of MS2-SA VLPs

Biotinylated MS2 was added dropwise to a molar excess of concentrated SA solution that was stirred vigorously in a 5-mL glass vial. After a 30-minute incubation, the MS2-SA VLP was separated from the excess SA through SEC with a Superdex 200 Increase 10/300 column (Cytiva). The MS2-SA VLP was quantified by boiling a small aliquot at 90 °C in Nu-PAGE lithium dodecyl sulfate (LDS) sample buffer (Invitrogen) for 30 min and running the sample on a polyacrylamide gel. SA standards with known concentrations quantified by UV absorption at 280 nm were also run on the gel. Comparing the intensities of the bands resulting from the SA standards with the intensity of the band representing the SA from the MS2-SA allowed for quantification of the VLP.

### Expression and purification of SARS-CoV-2 S proteins

DNA encoding the S-2P^13^ and HexaPro^18^ prefusion-stabilized versions of the SARS-CoV-2 S ectodomain (residues 1–1208) with a C-terminal T4 fibritin trimerization motif, AviTag, and a his-tag were cloned into pcDNA3.1 between the NcoI and XhoI restriction sites by Gene Universal Inc. (Newark, DE). These plasmids were transfected into Expi293F cells (Thermo Fisher Scientific) using the ExpiFectamine Transfection Kit and protocol (Thermo Fisher Scientific). Five days after transfection, the cells were pelleted by centrifugation for 20 min at 5500 × *g*. The supernatant was dialyzed into PBS and passed through 1 mL of HisPur Ni-NTA resin (Thermo Fisher Scientific) in a gravity flow column (G-Biosciences). The column was then washed with 40 mL of wash buffer (42 mM sodium bicarbonate, 8 mM sodium carbonate, 300 mM NaCl, 20 mM imidazole). The S proteins were eluted from the column by incubating the Ni-NTA resin with 3 mL of elution buffer (42 mM sodium bicarbonate, 8 mM sodium carbonate, 300 mM NaCl, 300 mM imidazole) for 5 min before allowing for flow by gravity. This elution procedure was repeated twice, resulting in 9 mL of eluate. The eluate was concentrated by using a 10-kDa MWCO centrifugal filter (Millipore Sigma). S proteins were buffer exchanged into 20 mM Tris, 20 mM NaCl, pH 8.0 to allow for in vitro biotinylation and were quantified by using the BCA assay (Thermo Scientific).

### In vitro biotinylation of AviTagged MS2 and SARS-CoV-2 S

Biotinylation was performed in vitro using a BirA biotin-protein ligase standard reaction kit (Avidity) following the manufacturer’s protocol. In brief, the protein solution (either MS2 or SARS-CoV-2 S) was buffer exchanged into a 20 mM Tris, 20 mM NaCl, pH 8.0 buffer and the protein concentration was adjusted to 45 µM. BirA and a proprietary mixture containing biotin, ATP, and magnesium acetate (Biomix B) was added to the protein solution. This solution was shaken vigorously at 37 °C. After 2 h at 37 °C, more Biomix B was added, and the solution was nutated at 4 °C overnight. The proteins of interest were then purified through SEC with a Superdex 200 Increase 10/300 column (Cytiva) connected to an ÄKTA pure (Cytiva) and controlled by Unicorn 7.2 software (Cytiva). Biotinylated S proteins were quantified by using the BCA assay (Thermo Scientific).

### Expression and purification of CR3022 and ACE2-Fc

The variable regions of the heavy and light chains of CR3022^17^ were cloned into the TGEX-HC and TGEX-LC vectors (Antibody Design Labs), respectively, according to the manufacturer’s protocol. Likewise, ACE2 (residues 1–615) was cloned into TGEX-HC. The DNA was then transfected into Expi293F cells (Thermo Fisher Scientific) by using the ExpiFectamine Transfection Kit (Thermo Fisher Scientific) following the provided protocol, and the cells were incubated in a humidified incubator at 37 °C and 8% CO_2_ for 5 days. The cells were then centrifuged at 5500 × *g* for 20 min. The supernatant media was diluted twofold in PBS and run through a 1-mL MabSelect SuRe column (Cytiva) connected to an ÄKTA start (Cytiva) and controlled by Unicorn start 1.0 software (Cytiva) according to the manufacturer’s operation manual to purify the proteins. CR3022 and ACE2-Fc were quantified by using the BCA assay (Thermo Scientific).

### SDS-PAGE

Protein samples were diluted fourfold in Nu-PAGE lithium dodecyl sulfate (LDS) sample buffer (Invitrogen). The samples were then boiled at 90 °C for 30 min. PageRuler Plus Prestained Protein Ladder (Thermo Scientific) and protein samples were pipetted into the wells of a 4–12% Bis-Tris gel (Invitrogen), which was run in MES-SDS buffer at 4 °C for 1 h at 110 V. The gel was stained with SimplyBlue SafeStain (Invitrogen) and subsequently de-stained. Once sufficiently de-stained, the gel was imaged by using the ChemiDoc MP imaging system and Image Lab 5.2.1 software (Bio-Rad).

### Preparation of VLP-S

MS2-SA and biotinylated S protein were mixed in a stoichiometric ratio found by using analytical SEC. We used analytical SEC to characterize mixtures consisting of 5 µg of biotinylated S protein and varying amounts of MS2-SA VLP. The ratio of the mixture that contained the least MS2-SA VLP and also did not have excess S protein appear on the chromatogram was the stoichiometric ratio used to create the VLP-S. The concentration of the VLP-S was adjusted such that the solution contained 0.12 µg of S per µL. The VLP-S were further characterized by use of ELISA, SEC, and DLS as described below.

### Characterization of S and VLP-S by ELISA

VLP-S and S protein in PBS were coated onto a Nunc Maxisorp 96-well plate such that each well contained 0.1 µg of S protein in 100 µL. After 1 h, the protein solutions were discarded from the wells and each well was blocked with 200 µL of 5% BSA (EMD Millipore) in PBST (PBS with 0.05% Tween-20) for 45 min. The plate was then washed twice with PBST, and CR3022 and ACE2-Fc in 1% BSA in PBST were added to the appropriate wells such that each well contained either one CR3022 or ACE2-Fc molecule per S trimer. One hour later, the wells were washed twice with PBST and a horseradish peroxidase-conjugated anti-human IgG Fc fragment goat antibody (MP Biomedicals; 1:5000 dilution) in 1% BSA in PBST was added to each well and left to incubate for 1 h. Then, the plate was washed twice with PBST and developed with TMB substrate solution (Thermo Scientific) for 3 min; the reaction was then stopped with 0.16 M sulfuric acid. The absorbance of each well at 450 nm was read using a Spectramax i3x plate reader (Molecular Devices) and Gen5 2.07 software (BioTek).

### Analytical SEC

A Superdex 200 Increase 10/300 column (Cytiva) connected to an ÄKTA pure (Cytiva) and controlled by Unicorn 7.2 software (Cytiva) was equilibrated with PBS. The 1-mL sample loop was washed with PBS and then 950 µl of either VLP-S solution or S alone was loaded into the sample loop. Each sample included 5 µg of S protein. The sample loop was then flushed with PBS such that the sample was directed through the column at a flowrate of 0.5 mL/min. One column volume of PBS was run through the column. Unicorn 7 (Cytiva) was used to control the system and to output a chromatogram of UV absorbance at 210 nm.

### Dynamic light scattering

A UVette (Eppendorf) containing 100 µL of VLP-S at a concentration of ~0.05 μg S per μL was loaded into a DynaPro NanoStar Dynamic Light Scattering detector (Wyatt Technology). For each measurement, Dynamics software (Wyatt Technology) was used to allow the temperature to equilibrate to 25 °C, to collect ten acquisitions, and to output the results. Results were displayed by % Mass using the Isotropic Spheres model.

### Negative stain transmission electron microscopy

Conventional negative-stain transmission electron microscopy (TEM) was performed, as described previously^28^. Briefly, 4 μl of the diluted samples was applied onto glow-discharged 300 mesh copper grids (CF300-Cu; Electron Microscopy Sciences, PA), washed with PBS (1X), and stained in droplets of 1% phosphotungstic acid (PTA, PH 6~7) for 1 min. The grids were then drained from the grid backside and air-dried inside a petri dish for at least 30 min at room temperature to minimize the negative-stain artifacts of flattening and stacking^29^.

### Plunge freezing and cryo-electron microscopy

4 μl of the VLP suspension was added to a glow discharged copper grid (C-Flat 1.2/1.3, 400 mesh, Protochips). Grids were plunged frozen into liquid ethane by double-sided blotting using Vitrobot Mark IV (ThermoScientific) and stored in liquid nitrogen until imaging. Cryo-electron microscopy (cryo-EM) and cryo-electron tomography (cryo-ET) were performed as described previously on a Titan Krios (ThermoScientific Hillsboro, OR, USA) at 300 kV^30^. Images (defocus of −5 μm) were recorded on a post-GIF Gatan K3 camera in EFTEM mode (4.603 Å/pixel) with a 20-eV slit, CDS counting mode, using SerialEM 3.8^31^. A total dose of 25-30 e/ Å^2^ was used and 34 frames were saved (1.14 e/ Å^2^ per frame). Frames were motion-corrected in MotionCor2^32^. Images were low pass filtered to 10 Å for better visualization and contrast in EMAN2.2^33^.

### Virus and titration assays

The virus isolate SARS-CoV-2/UT-NCGM02/Human/2020/Tokyo was used in this study and was previously characterized in Syrian hamsters^19^.

Virus titrations were performed on Vero E6/TMPRSS2 cells that were obtained from the National Institute of Infectious Diseases, Japan^34^. Cells were maintained in Dulbecco’s modified Eagle’s medium (DMEM) containing 10% fetal bovine serum (FBS) and antibiotic/antimycotic solution along with G418 (1 mg/ml).

To determine virus titers, confluent Vero E6/TMPRSS2 cells were infected with 100 µl of undiluted or 10-fold dilutions (10^−1^ to 10^−5^) of clarified lung or nasal turbinate homogenates. After a 30-minute incubation, the inoculum was removed, the cells were washed once, and then overlaid with 1% methylcellulose solution in DMEM with 5% FBS. The plates were incubated for three days, and then the cells were fixed and stained with 20% methanol and crystal violet to count the plaques.

### Hamster immunization study

The immunization study in hamsters was performed after approval by the Institutional Animal Care and Use Committee at the University of Wisconsin. Golden Syrian hamsters (4-week-old females) were immunized with either 60 µg of SARS-CoV-2 S protein presented on the MS2-SA VLP, an equal amount of MS2-SA VLP without the S protein, or an equal volume of sterile phosphate-buffered saline (PBS) by subcutaneous inoculation. Alhydrogel (2% solution; InvivoGen) added at an equal volume was thoroughly mixed with each vaccine preparation before inoculation. Animals were infected by intranasal inoculation with 10^3^ plaque-forming units (PFU) of SARS-CoV-2 while under isoflurane anesthesia. Animals were monitored daily for signs of illness and their body weights were recorded daily. Three days after infection, the animals were humanely sacrificed, and lung tissue and nasal turbinate samples were collected.

Serum was isolated from blood samples collected via the sublingual vein before the immunization and challenge with virus.

### Detection of antibodies against the RBD of SARS-CoV-2 S in immunized hamsters by ELISA

The ELISA was performed using a recombinant SARS-CoV-2 S RBD protein produced in Expi293F cells (Thermo Fisher Scientific) and then C-terminal his-tag purified by using TALON metal affinity resin. ELISA plates were coated overnight at 4 °C with 50 µl of the RBD protein at a concentration of 2 µg/ml in PBS. After being blocked with PBS containing 0.1% Tween 20 (PBS-T) and 3% milk powder, the plates with incubated in duplicate with heat-inactivated serum diluted in PBS-T with 1% milk powder. Goat anti-hamster IgG secondary antibody conjugated with horseradish peroxidase (Invitrogen; 1:7000 dilution) was used for detection. Plates were developed with SigmaFast o-phenylenediamine dihydrochloride solution (Sigma), and the reaction was stopped with the addition of 3 M hydrochloric acid. The absorbance was measured at a wavelength of 490 nm (OD_490_). Background absorbance measurements from serum collected before immunization were subtracted from the absorbance measurements from plasma collected before challenge for each dilution. IgG antibody endpoint titers were defined as the highest plasma dilution with an OD_490_ cut-off value of 0.15.

### Neutralization assay

Virus (~100 PFU) was incubated with the same volume of two-fold dilutions of heat-inactivated serum for 30 min at 37 °C. The antibody/virus mixture was added to confluent Vero E6/TMPRSS2 cells that were plated at 30,000 cells per well the day prior in 96-well plates. The cells were incubated for 3 days at 37 °C and then fixed and stained with 20% methanol and crystal violet solution. Virus neutralization titers were determined as the reciprocal of the highest serum dilution that completely prevented cytopathic effects.

### Biosafety statement

Research with SARS-CoV-2 was approved by the University of Wisconsin-Madison’s Institutional Biosafety Committee and performed under biosafety level 3 agriculture (BSL-3Ag) containment at the Influenza Research Institute, University of Wisconsin-Madison. The laboratory is approved for such use by the Centers for Disease Control and Prevention. The BSL-3Ag facility used was designed to exceed the standards outlined in *Biosafety in Microbiological and Biomedical Laboratories* (5th edition).

### Statistics and reproducibility

In vitro characterizations of the binding of Fc-ACE2 and CR3022 to the VLP-S constructs using ELISA (Figs. 2e and  3f) were each conducted twice independently with three technical replicates for each condition. The data are presented as the mean ± SD. For in vivo characterization, there were four groups (receiving either VLP-S, VLP-S, MS2-SA, or PBS) each with three hamsters (*n* = 3). To determine the resulting RBD IgG Endpoint Titers and Neutralizing Antibody titers (Table 1), three independent assays were conducted using sera from each hamster. The data are presented for each independent assay and also as the geometric mean with the geometric SD factor. Bodyweight after challenge with SARS-CoV-2 (Fig. 4b) was presented as the mean ± SD and significance was determined by a one-way analysis of variance (ANOVA) and Dunnett post-hoc multiple comparison between groups (α = 0.1). Assumptions of the normality of residuals and homogeneity of variance were validated by the D’Agostino-Pearson test and the Brown-Forsythe test, respectively. Viral titers in the lungs and nasal turbinates of hamsters immunized with either PBS, MS2-SA VLP, VLP-S_2Pro_ or VLP-S_6Pro_ 3 days after SARS-CoV-2 infection (Fig. 4c, d) were presented as the geometric mean with geometric SD (*n* = 3) and significance was determined by a one-way analysis of variance (ANOVA) and Dunnett post-hoc multiple comparison between groups (α = 0.1). Assumptions of the normality of residuals and homogeneity of variance were validated by the Shapiro-Wilk test and the Brown-Forsythe test, respectively. All statistical analysis was carried out using Excel 2013 (Microsoft) and Prism 8 (GraphPad).

### Reporting summary

Further information on research design is available in the Nature Research Reporting Summary linked to this article.

## Supplementary information

Supplementary Information

Description of Additional Supplementary Files

Supplementary Data

Reporting Summary

## Data Availability

The data needed to support the conclusions of this study have been included in the paper. The data that underly the graphs and charts within the paper are included in Supplementary Data 1. All other data supporting the conclusions of this study are available from the corresponding authors upon reasonable request.
