# Supplementary material for: Multivalent nanoparticle-based vaccines protect hamsters against SARS-CoV-2 after a single immunization
Source: Commun Biol. 2021 May 19;4:597. doi: 10.1038/s42003-021-02128-8 (PMC8134492; doi:10.1038/s42003-021-02128-8)
Supplement: Supplementary file 1 — Supplementary Information [file 42003_2021_2128_MOESM1_ESM.pdf]

Supplementary Information

Multivalent Nanoparticle-Based Vaccines Protect Hamsters Against SARS-CoV-2 After a Single Immunization

Shiho Chiba[1,†], Steven J. Frey[2,†], Peter J. Halfmann[1], Makoto Kuroda[1], Tadashi Maemura[1], Jie E. Yang[3,4], Elizabeth R. Wright[3,4], Yoshihiro Kawaoka[1,5]*, Ravi S. Kane[2,]*

[1]Influenza Research Institute, Department of Pathobiological Sciences, School of Veterinary Medicine, University of Wisconsin, Madison, WI, 53711, USA

[2]School of Chemical & Biomolecular Engineering, Georgia Institute of Technology, Atlanta, Georgia, 30332, USA

[3]Department of Biochemistry, University of Wisconsin, Madison, WI, 53706, USA

[4]Cryo-EM Research Center, Department of Biochemistry, University of Wisconsin, Madison, WI, 53706, USA

[5]Division of Virology, Department of Microbiology and Immunology, Institute of Medical Science, University of Tokyo, Tokyo 108-8639, Japan

† These authors contributed equally

* To whom correspondence should be addressed:
yoshihiro.kawaoka@wisc.edu, ravi.kane@chbe.gatech.edu

Supplementary Figures

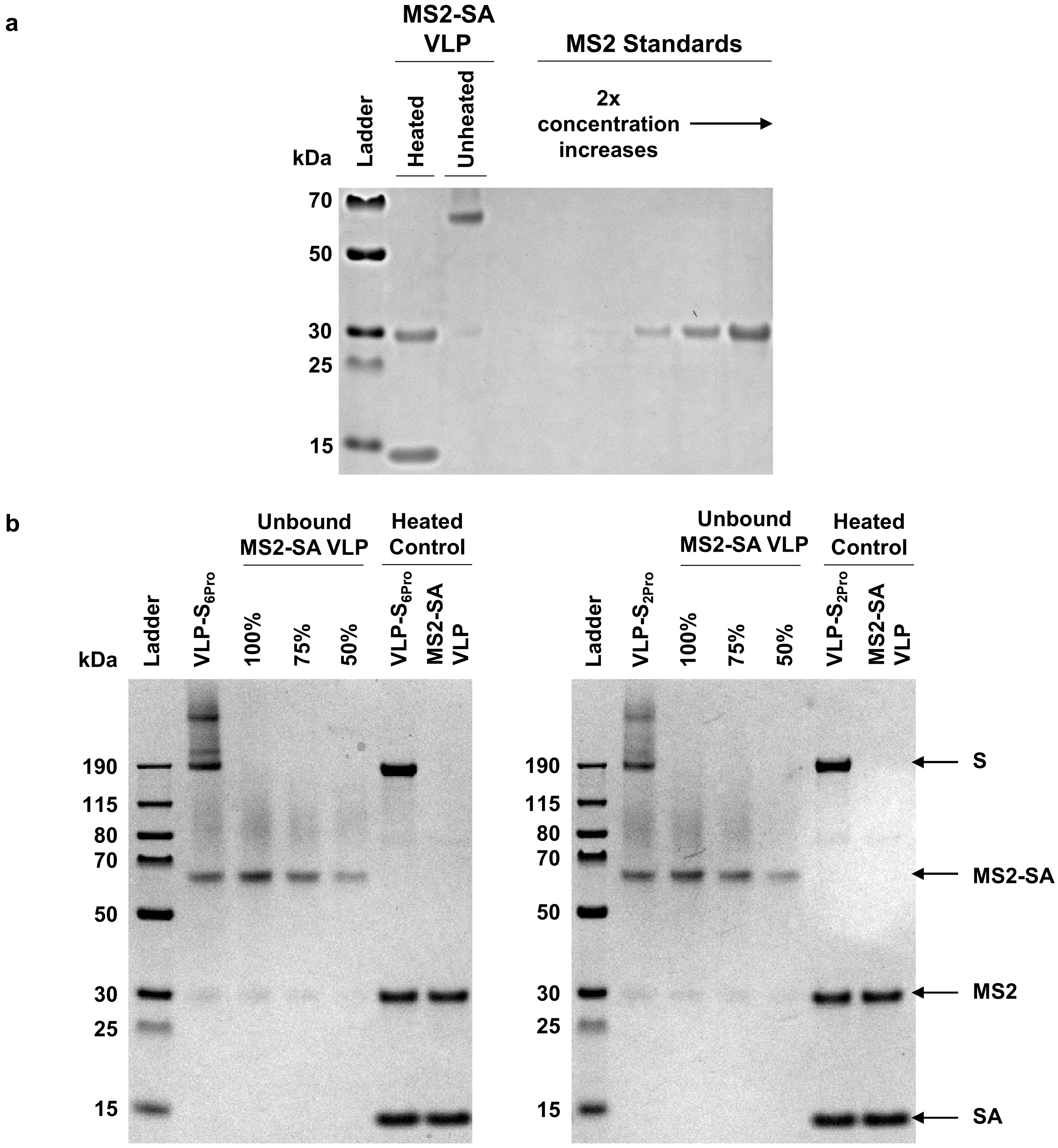

Supplementary Figure 1. Characterization of MS2 to SA to S stoichiometry using SDS-PAGE. a) Amount of MS2 in heated MS2-SA VLP was compared to amount of MS2 in unheated MS2-SA VLP to determine that approximately 80 percent of MS2 is occupied by SA. Excess biotin was added to the unheated sample to occupy all unoccupied biotin binding sites prior to the addition of SDS. β-mercaptoethanol was added to all samples. b) Amount of unbound MS2-SA in VLP-S was compared to MS2-SA VLP standards to determine approximately 25 percent of MS2-SA in VLP-S was occupied by S. A heated control was included to ensure the same amount of VLP was present within the VLP-S and 100% VLP standard. Excess biotin was added to the unheated samples to occupy all unoccupied biotin binding sites prior to the addition of SDS. β-mercaptoethanol was added to all samples. The unprocessed gels are shown in Supplementary Figure 3.

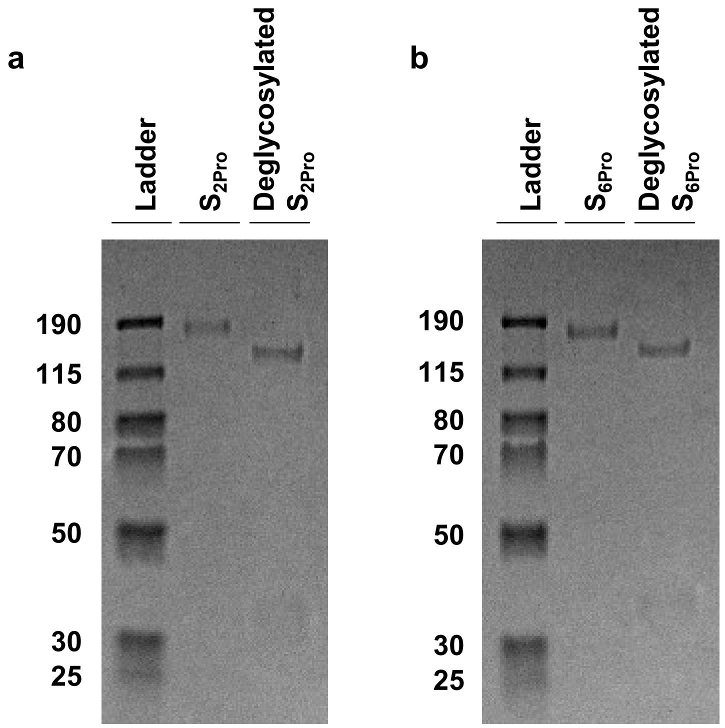

Supplementary Figure 2. SDS-PAGE gels of deglycosylated S proteins. S_{2Pro} (a) and S_{6Pro} (b) before and after deglycosylation with PNGase F. The unprocessed gel is shown in Supplementary Figure 3.

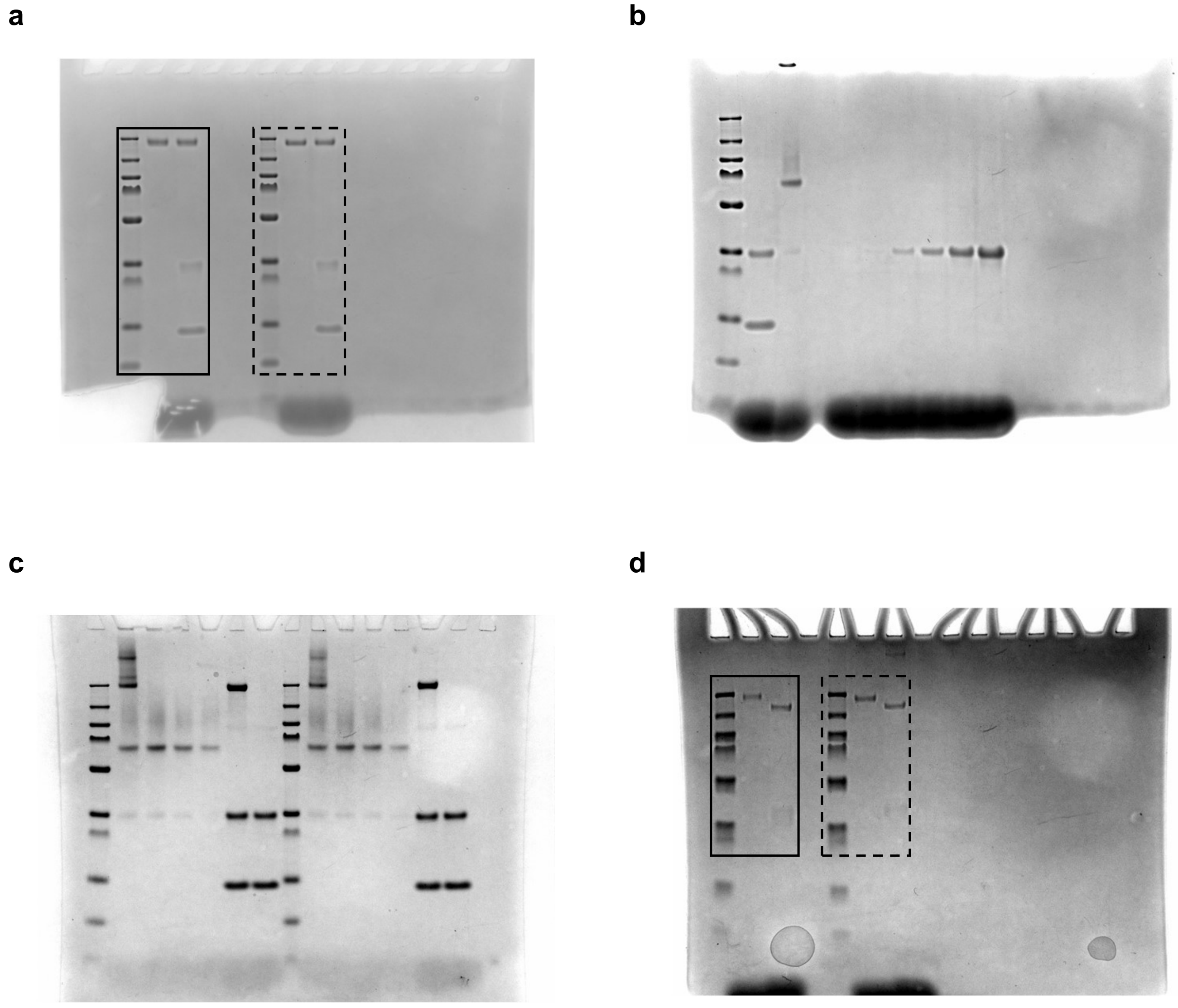

Supplementary Figure 3. Unprocessed SDS-PAGE gel images. Unprocessed SDS-PAGE gel images, cropped versions of which appear in (a) Figure 2a (solid rectangle), Figure 3a (dashed rectangle), (b) Supplementary Figure 1a, (c) Supplementary Figure 1b, (d) Supplementary Figure 2a (solid rectangle), and Supplementary Figure 2b (dashed rectangle).

Supplementary Notes

Supplementary Note 1. MS2-AviTag Sequence

MASNFTQFVLVDNGGTGDVTVAPSNFANGVAEWISSNSRSQAYKVTCSVRQSSAQNRKYTIKVEVPKVATQTVG
GVELPVAAWRSYLNMELTIPIFATNSDCELIVKAMQGLLKDGNPIPSAIAANSGIYASNFTQFVLVDNGGGLND
IFEAQKIEWHETGDVTVAPSNFANGVAEWISSNSRSQAYKVTCSVRQSSAQNRKYTIKVEVPKVATQTVGGVEL
PVAAWRSYLNMELTIPIFATNSDCELIVKAMQGLLKDGNPIPSAIAANSGIY

Supplementary Note 2. S_{2Pro} Sequence

MFVFLVLLPLVSSQCVNLTTRTQLPPAYTNSFTRGVYYPDKVFRSSVLHSTQDLFLPFFSNVTWFHAIHVSGTN
GTKRFDNPVLPFNDGVYFASTEKSNIIRGWIFGTTLDSKTQSLLIVNNATNVVIKVCEFQFCNDPFLGVYYHKN
NKSWMESEFRVYSSANNCTFEYVSQPFLMDLEGKQGNFKNLREFVFKNIDGYFKIYSKHTPINLVRDLPQGFSA
LEPLVDLPIGINITRFQTLLALHRSYLTPGDSSSGWTAGAAAYYVGYLQPRTFLLKYNENGTITDAVDCALDPL
SETKCTLKSFTVEKGIYQTSNFRVQPTESIVRFPNITNLCPFGEVFNATRFASVYAWNRKRISNCVADYSVLYN
SASFSTFKCYGVSPTKLNDLCFTNVYADSFVIRGDEVRQIAPGQTGKIADYNYKLPDDFTGCVIAWNSNNLDSK
VGGNYNYLYRLFRKSNLKPFERDISTEIYQAGSTPCNGVEGFNCYFPLQSYGFQPTNGVGYQPYRVVVLSFELL
HAPATVCGPKKSTNLVKNKCVNFNFNGLTGTGVLTESNKKFLPFQQFGRDIADTTDAVRDPQTLEILDITPCSF
GGVSVITPGTNTSNQVAVLYQDVNCTEVPVAIHADQLTPTWRVYSTGSNVFQTRAGCLIGAEHVNNSYECDIPI
GAGICASYQTQTNSPGSASSVASQSIIAYTMSLGAENSVAYSNNSIAIPTNFTISVTTEILPVSMTKTSVDCTM
YICGDSTECSNLLLQYGSFCTQLNRALTGIAVEQDKNTQEVFAQVKQIYKTPPIKDFGGFNFSQILPDPSKPSK
RSFIEDLLFNKVTLADAGFIKQYGDCLGDIAARDLICAQKFNGLTVLPPLLTDEMIAQYTSALLAGTITSGWTF
GAGAALQIPFAMQMAYRFNGIGVTQNVLYENQKLIANQFNSAIGKIQDSLSSTASALGKLQDVVNQNAQALNTL
VKQLSSNFGAISSVLNDILSRLDPPEAEVQIDRLITGRLQSLQTYVTQQLIRAAEIRASANLAATKMSECVLGQ
SKRVDFCGKGYHLMSFPQSAPHGVVFLHVTYVPAQEKNFTTAPAICHDGKAHFPREGVFVSNGTHWFVTQRNFY
EPQIITTDNTFVSGNCDVVIGIVNNTVYDPLQPELDSFKEELDKYFKNHTSPDVDLGDISGINASVVNIQKEID
RLNEVAKNLNESLIDLQELGKYEQGSGYIPEAPRDGQAYVRKDGEWVLLSTFLGGLNDIFEAQKIEWHEHHHHH
H

Supplementary Note 3. S_{6Pro} Sequence

MFVFLVLLPLVSSQCVNLTTRTQLPPAYTNSFTRGVYYPDKVFRSSVLHSTQDLFLPFFSNVTWFHAIHVSGTN
GTKRFDNPVLPFNDGVYFASTEKSNIIRGWIFGTTLDSKTQSLLIVNNATNVVIKVCEFQFCNDPFLGVYYHKN
NKSWMESEFRVYSSANNCTFEYVSQPFLMDLEGKQGNFKNLREFVFKNIDGYFKIYSKHTPINLVRDLPQGFSA
LEPLVDLPIGINITRFQTLLALHRSYLTPGDSSSGWTAGAAAYYVGYLQPRTFLLKYNENGTITDAVDCALDPL
SETKCTLKSFTVEKGIYQTSNFRVQPTESIVRFPNITNLCPFGEVFNATRFASVYAWNRKRISNCVADYSVLYN
SASFSTFKCYGVSPTKLNDLCFTNVYADSFVIRGDEVRQIAPGQTGKIADYNYKLPDDFTGCVIAWNSNNLDSK
VGGNYNYLYRLFRKSNLKPFERDISTEIYQAGSTPCNGVEGFNCYFPLQSYGFQPTNGVGYQPYRVVVLSFELL
HAPATVCGPKKSTNLVKNKCVNFNFNGLTGTGVLTESNKKFLPFQQFGRDIADTTDAVRDPQTLEILDITPCSF
GGVSVITPGTNTSNQVAVLYQDVNCTEVPVAIHADQLTPTWRVYSTGSNVFQTRAGCLIGAEHVNNSYECDIPI
GAGICASYQTQTNSPGSASSVASQSIIAYTMSLGAENSVAYSNNSIAIPTNFTISVTTEILPVSMTKTSVDCTM
YICGDSTECSNLLLQYGSFCTQLNRALTGIAVEQDKNTQEVFAQVKQIYKTPPIKDFGGFNFSQILPDPSKPSK
RSPIEDLLFNKVTLADAGFIKQYGDCLGDIAARDLICAQKFNGLTVLPPLLTDEMIAQYTSALLAGTITSGWTF
GAGPALQIPFPMQMAYRFNGIGVTQNVLYENQKLIANQFNSAIGKIQDSLSSTPSALGKLQDVVNQNAQALNTL
VKQLSSNFGAISSVLNDILSRLDPPEAEVQIDRLITGRLQSLQTYVTQQLIRAAEIRASANLAATKMSECVLGQ
SKRVDFCGKGYHLMSFPQSAPHGVVFLHVTYVPAQEKNFTTAPAICHDGKAHFPREGVFVSNGTHWFVTQRNFY
EPQIITTDNTFVSGNCDVVIGIVNNTVYDPLQPELDSFKEELDKYFKNHTSPDVDLGDISGINASVVNIQKEID
RLNEVAKNLNESLIDLQELGKYEQGSGYIPEAPRDGQAYVRKDGEWVLLSTFLGGLNDIFEAQKIEWHEHHHHH
H
