# Supplementary material for: Multivalent nanoparticle-based vaccines protect hamsters against SARS-CoV-2 after a single immunization
Source: Commun Biol. 2021 May 19;4:597. doi: 10.1038/s42003-021-02128-8 (PMC8134492; doi:10.1038/s42003-021-02128-8)
Supplement: Supplementary file 2 — Description of Additional Supplementary Files [file 42003_2021_2128_MOESM2_ESM.pdf]

Description of Additional Supplementary Files

File name: Supplementary Data

Description: Source data for VLP-S characterization ELISAs (Figure 2e and Figure 3f), body weight of immunized hamsters (Figure 4b), and viral titer in lungs (Figure 4c) and nasal turbinates (Figure 4d) of immunized hamsters.
