# Supplementary material for: Multivalent nanoparticle-based vaccines protect hamsters against SARS-CoV-2 after a single immunization
Source: Commun Biol. 2021 May 19;4:597. doi: 10.1038/s42003-021-02128-8 (PMC8134492; doi:10.1038/s42003-021-02128-8)
Supplement: Supplementary file 4 — Reporting Summary [file 42003_2021_2128_MOESM4_ESM.pdf]

nature research

Corresponding author(s): Dr. Ravi S. Kane
Dr. Yoshihiro Kawaoka

Last updated by author(s): Apr 9, 2021

Reporting Summary

Nature Research wishes to improve the reproducibility of the work that we publish. This form provides structure for consistency and transparency in reporting. For further information on Nature Research policies, see our Editorial Policies and the Editorial Policy Checklist.

Statistics

For all statistical analyses, confirm that the following items are present in the figure legend, table legend, main text, or Methods section.

n/a	Confirmed	
☐	☒	The exact sample size (n) for each experimental group/condition, given as a discrete number and unit of measurement
☐	☒	A statement on whether measurements were taken from distinct samples or whether the same sample was measured repeatedly
☐	☒	The statistical test(s) used AND whether they are one- or two-sided<br>*Only common tests should be described solely by name; describe more complex techniques in the Methods section.*
☒	☐	A description of all covariates tested
☐	☒	A description of any assumptions or corrections, such as tests of normality and adjustment for multiple comparisons
☐	☒	A full description of the statistical parameters including central tendency (e.g. means) or other basic estimates (e.g. regression coefficient) AND variation (e.g. standard deviation) or associated estimates of uncertainty (e.g. confidence intervals)
☐	☒	For null hypothesis testing, the test statistic (e.g. F, t, r) with confidence intervals, effect sizes, degrees of freedom and P value noted<br>*Give P values as exact values whenever suitable.*
☒	☐	For Bayesian analysis, information on the choice of priors and Markov chain Monte Carlo settings
☒	☐	For hierarchical and complex designs, identification of the appropriate level for tests and full reporting of outcomes
☒	☐	Estimates of effect sizes (e.g. Cohen's d, Pearson's r), indicating how they were calculated

Our web collection on statistics for biologists contains articles on many of the points above.

Software and code

Policy information about availability of computer code

Data collection	UNICORN 7.2 (Cytiva), UNICORN start 1.0 (Cytiva), DYNAMICS 7.0.1 (Wyatt Technology), Image Lab 5.2.1 (Bio-Rad), Gen5 2.07 (BioTek)
Data analysis	Prism 8 (GraphPad), Excel 2013 (Microsoft)

For manuscripts utilizing custom algorithms or software that are central to the research but not yet described in published literature, software must be made available to editors and reviewers. We strongly encourage code deposition in a community repository (e.g. GitHub). See the Nature Research guidelines for submitting code & software for further information.

Data

Policy information about availability of data

All manuscripts must include a data availability statement. This statement should provide the following information, where applicable:

- Accession codes, unique identifiers, or web links for publicly available datasets
- A list of figures that have associated raw data
- A description of any restrictions on data availability

We have included a data availability statement

Field-specific reporting

Life sciences study design

All studies must disclose on these points even when the disclosure is negative.

Sample size	We did not predetermine sample size using software. The exact n values used to calculate the statistics are provided and a reasonable sample size was chosen to ensure adequate reproducibility of results.
Data exclusions	No data were excluded from the analysis.
Replication	Replicates were used in all experiments as described.
Randomization	Hamsters were randomly assigned to groups.
Blinding	Blinding was not performed, as this experiment involves measurements that are not subjective and data analysis that is performed systematically.

Reporting for specific materials, systems and methods

We require information from authors about some types of materials, experimental systems and methods used in many studies. Here, indicate whether each material, system or method listed is relevant to your study. If you are not sure if a list item applies to your research, read the appropriate section before selecting a response.

Materials & experimental systems

n/a	Involved in the study	
☐	☒	Antibodies
☐	☒	Eukaryotic cell lines
☒	☐	Palaeontology and archaeology
☐	☒	Animals and other organisms
☒	☐	Human research participants
☒	☐	Clinical data
☒	☐	Dual use research of concern

Methods

n/a	Involved in the study	
☒	☐	ChIP-seq
☒	☐	Flow cytometry
☒	☐	MRI-based neuroimaging

Antibodies

Antibodies used	CR3022 was generated as described in the methods section. HRP-conjugated anti-human IgG (Fc fragment specific) affinity-purified polyclonal goat antibody (secondary) is supplied by MP Biomedicals (Cat# 674171, Lot# S4456). Goat anti-hamsters IgG (H+L) Secondary Antibody, HRP is supplied by ThermoFisher (Cat# PA1-29626).
Validation	The purity of CR3022 was analyzed using SDS-PAGE, and its ability to bind to SARS-CoV-2 Spike and receptor binding domain proteins was verified using ELISA.

Eukaryotic cell lines

Policy information about cell lines

Cell line source(s)	Vero E6/TMPRSS2 cells were generated by the National Institute of Infectious Diseases; Tokyo Japan (DOI: https://doi.org/10.1073/pnas.2002589117). Expi293F cells were supplied by Thermo Fisher Scientific and first described by Jones et al. (DOI: 10.1089/gen.32.17.21).
Authentication	Vero E6/TMPRSS2 cells were not authenticated, but infection with SARS-CoV-2 was consistent with expectations. Expi293F was not authenticated, but cell morphology, growth, viability, and protein expression was consistent with expectations.
Mycoplasma contamination	Vero E6/TMPRSS2 cells were tested monthly for mycoplasma and were negative at all times.
Commonly misidentified lines (See ICLAC register)	No commonly misidentified cell lines were used in this study.

Animals and other organisms

Policy information about studies involving animals; ARRIVE guidelines recommended for reporting animal research

Laboratory animals	Syrian golden hamsters (4-week old females) were obtained from Envigo (Indianapolis, IN).
Wild animals	N/A

Field-collected samples	N/A
Ethics oversight	The immunization study in hamsters was performed after approval by the Institutional Animal Care and Use Committee at the University of Wisconsin.

Note that full information on the approval of the study protocol must also be provided in the manuscript.
